# Adherence with cardiovascular medications and the outcomes in patients with coronary arterial disease: “Real‐world” evidence

**DOI:** 10.1002/clc.23898

**Published:** 2022-09-18

**Authors:** Chen Chen, Xiaoqing Li, Yuhao Su, Zhigang You, Rong Wan, Kui Hong

**Affiliations:** ^1^ Department of Cardiovascular Medicine The Second Affiliated Hospital of Nanchang University Nanchang Jiangxi China; ^2^ Jiangxi Key Laboratory of Molecular Medicine Nanchang Jiangxi China; ^3^ Department of Genetic Medicine The Second Affiliated Hospital of Nanchang University Nanchang Jiangxi China

**Keywords:** ACEI/ARB, beta‐blocker, coronary heart disease, dose–response, medication adherence, risk factor, statin

## Abstract

**Background:**

Cardiovascular medications are vital for the secondary prevention of coronary arterial disease (CAD). However, the effect of cardiovascular medication may depend on the optimal adherence of the patients. This meta‐analysis aims to determine the magnitude of adherence to vascular medications that influences the absolute and relative risks (RRs) of mortality in patients with CAD in real‐world settings.

**Methods:**

The Cochrane Library, PubMed, and EMBASE databases were searched through March 1, 2022. Prospective studies reporting association as RR and 95% confidence interval between cardiovascular medication adherence and any cardiovascular events and/or all‐cause mortality in patients with CAD were included. A one‐stage robust error meta‐regression method was used to summarize the dose‐specific relationships.

**Results:**

A total of 18 studies were included. There is a significant inverse linear association between cardiovascular medication adherence and cardiovascular events (*p*_nonlinearity_ = .68) or mortality (*p*_nonlinearity_ = .82). The exposure‐effect analysis showed that an improvement of 20% cardiovascular medication adherence was associated with 8% or 12% lower risk of any cardiovascular events or mortality, respectively. In subgroup analysis, the benefit was observed in adherence of stain (RR: 0.90, for cardiovascular events, RR: 0.85, for mortality), angiotensin‐converting enzyme inhibitors (ACEI)/angiotensin II receptor blockers (ARB)(RR: 0.90, for mortality), and antiplatelet agent (RR: 0.89 for mortality) but not in beta‐blocker (RR: 0.90, *p* = .14, for cardiovascular events, RR: 0.97, *p* = .32 for mortality). Estimated absolute differences per 1 million individuals per year for mortality associated with 20% improvement were 175 cases for statin, 129 cases for antiplatelet, and 117 cases for ACEI/ARB.

**Conclusion:**

Evidence from the real word showed poor adherence to vascular medications contributes to a considerable proportion of all cardiovascular disease events and mortality in patients with CAD.

## 1 INTRODUCTION

Coronary arterial disease (CAD) is a highly prevalent disease, associated with increased costs, morbidity, and mortality.^1^ Cardiovascular medications, such as angiotensin‐converting enzyme inhibitos (ACEI)/angiotensin II receptor blockers (ARB), beta‐blockers, antiplatelet agents, and statins, remain the most common medical interventions worldwide for the prevention of CAD.^2^, ^3^, ^4^ Although their beneficial effects have been established in the primary and secondary prevention of cardiovascular diseases (CVDs), their effort in real‐world settings is inferior to that seen in random control trials (RCT), and this has been partly attributed to poor medication adherence. According to a recent study, almost 31% of myocardial infarction (MI) patients are no longer persistent with their prescribed medications by 6 months.^5^ A meta‐analysis that included 1 978 919 patients showed that only 60% of patients were adherence to their cardiovascular medications. Additionally, compared with good adherence, the risk of cardiovascular events or mortality in those with poor adherence increased by 20% or 35%, respectively.^6^

A systematic evaluation of the association between cardiovascular medication adherence and the outcomes in patients with CAD is of significance for understanding the role of medication adherence in secondary prevention. Furthermore, a quantitative analysis of adherence and their outcomes could provide more detailed guidelines and education information for patients with cardiovascular medication to have better outcomes (e.g., cardiovascular events and mortality). However, the quantitative association between cardiovascular medication adherence and long‐term outcomes in patients with CAD remains to be determined. Moreover, considering well‐designed RCTs might not reflect the actual adherence level or effectiveness of medication therapy in the “real” community.^7^ We conducted an exposure‐effect meta‐analysis based on prospective observational studies to (i) quantitatively investigate the relationship between adherence to cardiovascular medication and outcomes in patients with CAD in real‐world settings and (ii) estimate the future absolute risk for cardiovascular events or mortality for suboptimum adherence to cardiovascular medication.

## 2 METHODS

We conducted this meta‐analysis according to preferred reporting items for systematic reviews and meta‐analyses guidelines^8^ (Supporting Information: Table 1). All prospective studies (cohort, nested case‐control), reporting data about medication adherence (statin, antiplatelet agents, ACEI/ARB, and beta‐blockers) and any cardiovascular (defined as any fatal or nonfatal coronary heart disease) events, or all‐cause death were considered eligible for the systematic review. A comprehensive literature search was performed using Cochrane Library, PubMed, and Embase databases, up to March 1, 2022. Two researchers (Chen Chen and Xiaoqing Li) independently worked in the whole process of this meta‐analysis from the literature search and selection to data analysis. Supporting Information: Table S2 provides a detailed description of the search strategy. All discrepancies were resolved through discussion with each other or through consultation with a third reviewer (Yuhao Su). We used a robust error meta‐regression method^9^ for the exposure‐effect analysis of cardiovascular medication adherence and any cardiovascular events and all‐cause death. All statistical analyses were undertaken using Stata software (version 14.0; Stata Corp LP). Assessment of the quality of the included studies was performed using the Newcastle–Ottawa quality assessment scale (NOS), with a score over 6 defined as high quality.^10^ Full details of the literature search strategy, study selection criteria, quality assessment, and statistical analysis have been reported in Supporting Information: Methods. This study has been registered with PROSPERO (international prospective register of systematic reviews)–registration number–CRD42019116748.

**Table 1:** Basic characteristics of the 18 articles included in the meta‐analysis

Author, publication year, country	Baseline population	Population source	Design	Mean age (years), male (%)	Adherence measure	Medicine	Outcome
Allonen 2012, Finland	ACS	GPCAD study	Cohort	65.6, 69.6	MPR*	Statin	All‐cause death
Hamood 2015, Israel	Post‐AMI	LHS database	Cohort	66.36, 73.8	PDC	Statin Aspirin Beta‐blocker ACEI/ARB	All‐cause death
Ho 2006, USA	Diabetes and IHD	KPCO database	Cohort	69, 61	PDC	Statin Beta‐blocker ACEI/ARB	All‐cause mortality
Kleiner 2009, USA	Post‐AMI	Northeastern health database	Cohort	65.93, 62	PDC	Beta‐blocker	All‐cause mortality
Lenzi 2014, Italy	Post‐AMI	HDRs of LHA Database	Nested case‐control	77, 58.2	PDC	Statin Antiplatelet Beta‐blocker ACEI/ARB	All‐cause mortality
Martino 2015, Italy	Post‐MI	Regional hospital information system of Lazio	Nested case‐control	63.7 (men) 72.5 (women), 67.5	PDC	Statin Beta‐blocker ACEI/ARB	Reinfarction (mortality, or hospital admission for myocardial infarction [MI])
Rasmussen 2007, Canada	Post‐AMI	Ontario Myocardial Infarction Database	Cohort	≥66	PDC	Statin Beta‐blocker	All‐cause mortality
Rublee 2012, USA	CHD	I3 In Vision Data Mart insurance database,	Cohort	60.6, 64.6	PDC	Statin	Cardiovascular events
Ruokoniemi 2011, Finland	Diabetes with CHD	Administrative health databases in Finland	Nested case‐control	65.2, 68.9	PDC	Statin	Major coronary events
Tuppin 2010, France	Post‐MI	SNIIRAM‐PMSI	Cohort	NA	PDC	Statin Aspirin Beta‐blocker ACEI/ARB	Mortality or readmission for ACS
Shalev 2009, Israel	CHD	MHS	Cohort	61.5, 55.7	PDC	Statin	All‐cause mortality
Wei 2002, UK	Post‐MI	MEMO record‐linkage database 1985–1995	Cohort	67.26, 59.6	PDC	Statin	All‐cause mortality
WEI 2008, UK	Cardiovascular disease	MEMO record‐linkage database 1993–2001	Cohort	>65, 54.2	MPR	Statin Aspirin	All‐cause mortality recurrence of cardiovascular disease
Wei 2004, UK	Post‐MI	MEMO record‐linkage database 1994–1995	Cohort	66.3, 59.4	MPR	Beta‐blocker	All‐cause mortality re‐MI
Xie 2017, China	ACS	CPACS‐1 and CPACS‐2	Cohort	63.3, 70	PDC	Statin	All‐cause mortality, incident MI, stroke
McGinnis 2009, USA	Undergoing Coronary event	CPCRS 2000–2005	Cohort	62, 70	PDC	Statin	All‐cause mortality recurrent nonfatal cardiac events
Hickson 2019, USA	Post‐AMI	CMS chronic conditions database	Cohort	>65, 45.7	PDC	Statin	All‐cause mortality
Korhonen 2017, USA	Post‐AMI	CMS chronic condition database 2007–2011	Cohort	>65, 45.2	PDC	Statin Beta‐blocker ACEI/ARB	All‐cause mortality

Abbreviations: ACEI/ARB, angiotensin converter enzyme inhibitor/angiotensin receptor blocker; ACS, acute coronary syndrome; AMI, acute myocardial infarction; CABG, coronary artery bypass grafting; CCB, calcium channel blockers; CDS, chronic disease score; CHD, coronary heart disease; CHF, congestive heart failure; CKD, chronic kidney diseases; CMS, Medicare and Medicaid Services; COPD, chronic obstructive pulmonary disease; CPACS, clinical pathways for acute coronary syndromes in China; CPCRS, Kaiser Permanente Colorado and Clinical Pharmacy Cardiac Risk Service; ECG, electrocardiograph; GPCAD, genetic predisposition of coronary artery disease; HDRs, hospital discharge records; HF, heart failure; IHD, ischemic heart dieases; KPCO, Kaiser Permanente of Colorado Hospital; LHA, Local Health Authority; LHS, Leumit Health Services; MEMO, Medicine Monitoring Unit's; MHS, Maccabi Healthcare Services; MPR, medication possession ratio; NA, not applicable; NSTEMI, non‐ST‐elevation myocardial infarction; PCI, percutaneous coronary intervention; PDC, proportion of days covered; PVD, peripheral vascular disease; SNIIRAM‐PMSI, système national d'informations interrégimes de l'assurance maladie‐French hospital discharge database; TIA, transient ischemic attack.

*Categorized as no user (0), no‐regular user (poor adherence), and regular user (good adherence).

## 3 RESULTS

### 3.1 Study selection

**Figure 1:**
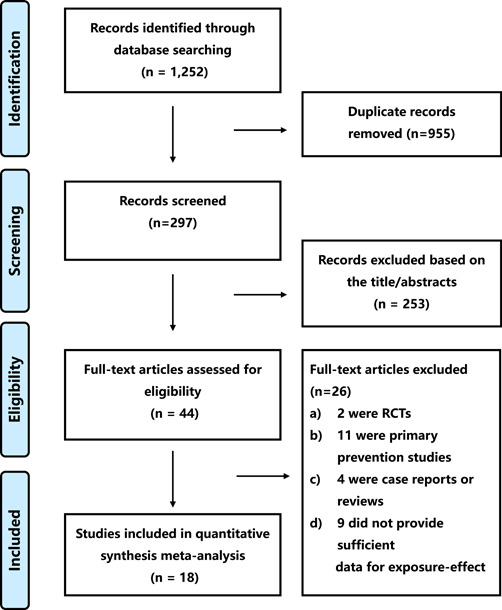
Flowchart of study selection. RCT, random control trial.

As shown in Figure 1, we initially identified studies in the Cochrane Library (*n* = 80), PubMed (*n* = 454), and Embase databases (*n* = 718). No additional studies were identified through manual searches. We excluded 955 studies based on screening the title or abstract, and the full text of the remaining 44 studies was reviewed. After a screening of the full‐text articles, 26 studies did not meet the selection criteria and were excluded (the detailed reason was listed in Figure 1). Finally, 18^11^, ^12^, ^13^, ^14^, ^15^, ^16^, ^17^, ^18^, ^19^, ^20^, ^21^, ^22^, ^23^, ^24^, ^25^, ^26^, ^27^, ^28^ prospective studies comprising 402 201 participants were considered potentially eligible and were included in the exposure‐effect analysis.

### 3.2 Study characteristics and quality

Detailed characteristics of the included studies are presented in Table 1. Overall, these studies were published between 2002 and 2022. The sample sizes of the included studies varied from 865 to 101 011 with a total of 402 201 individuals. The mean age ranged from 62 to 77 years. Most studies were designed as cohorts (*n* = 15) and three were nested case‐control. Seven were from North America (US and Canada); three were from Asia, and eight were from Europe.

The reporting quality of the included articles was high. All included studies obtained a NOS of ≥7 points (Supporting Information: Table S3).

### 3.3 Medication adherence and all‐cause death

Fifteen^11^, ^12^, ^13^, ^14^, ^15^, ^16^, ^17^, ^18^, ^19^, ^20^, ^23^, ^25^, ^26^, ^27^, ^28^ studies with available data on all‐cause mortality outcomes. Statin adherence was the most commonly studied (*n* = 13). There were five studies that assessed the ACEI/ARB drugs, eight assessed beta‐blockers, and 3 assessed antiplatelets, respectively.

**Figure 2:**
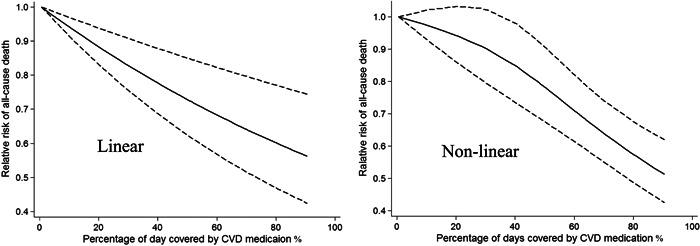
Dose–response analysis (linear and nonlinear) of cardiovascular medication adherence and all‐cause death. The solid line and the dashed lines represent the estimated relative risk and the 95% confidence interval, respectively. CVD, cardiovascular disease.

No evidence of nonlinear association was observed between cardiovascular medication adherence and all‐cause death (*p*_nonlinearity_ = .82). An improvement of 20% cardiovascular medication adherence was associated with a 12% lower risk (relative risk [RR]: 0.88; 95% CI: 0.82–0.97, *I* ^2^ = 19%, *p* < .001) for all‐cause death, with low evidence of heterogeneity (Figure 2). In the nonlinear model, an inverse association was found between cardiovascular medication adherence and all‐cause death (Figure 2). In subgroup analysis, a 20% adherence increment was associated with 15% (95% CI: 0.82–0.89, *I* ^2^ = 82%) lower mortality risk for statin, 11% (95% CI: 0.85–0.92, *I* ^2^ = 97%) for antiplatelet agents, and 10% (95% CI: 0.80–1.00, *I* ^2^ = 47%) for ACEI/ARB. However, the benefit was not observed in beta‐blocker (RR: 0.97. 95% CI: 0.93–1.03, *I* ^2^ = 97%).

The association between good adherence and mortality persisted in almost all subgroup analyses defined by age, region, follow‐up times, and other covariates (Supporting Information: Table S4).

### 3.4 Medication adherence and any CVD events

**Figure 3:**
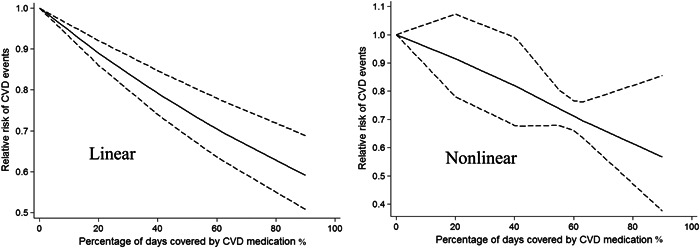
Dose–response relationship (linear and nonlinear) of cardiovascular medication adherence and cardiovascular events. The solid line and the dashed lines represent the estimated relative risk and the 95% confidence interval, respectively. CVD, cardiovascular disease.

Among six studies^19^, ^21^, ^22^, ^24^, ^25^, ^26^ reporting on cardiovascular events, statin adherence was the most commonly studied (*n* = 5). Two studies assessed the antiplatelet agents and two assessed beta‐blockers. Only one study was identified in the literature search that assessed the association of ACEI/ARB compliance with cardiovascular events. There was no evidence of a nonlinear relationship between cardiovascular medication adherence level and the risk of cardiovascular events (*p*_nonlinearity_ = .68). The exposure‐effect analysis showed an improvement in cardiovascular medication adherence of 20% was associated with an 8% lower risk of any cardiovascular events (RR: 0.92; 95% CI: 0.87–0.98, *I* ^2^ = 0%, *p* = .02), with no evidence of heterogeneity (Figure 3). In the nonlinear model, an inverse association was found between cardiovascular medication adherence and CVD events (Figure 3).

In subgroup analysis, there was also evidence of a linear association between statin (*p*_nonlinearity_ = .64) and beta‐blockers (*p*_nonlinearity_ = .48) and any cardiovascular events. The corresponding RRs were 0.90 (95% CI: 0.86–0.95, *I* ^2^ = 78%) for statin, 0.90 (95% CI: 0.67–1.21, *I* ^2^ = 0%) for beta‐blockers per 20% adherence increase, respectively. The subgroup analysis for antiplatelet and ACEI/ARB and antiplatelet agents was not available because of a limited number of studies.

### 3.5 Absolute risk difference associated with poor adherence

Using the cardiovascular events rate from the studies based on the US population,^29^ we estimated a 20% adherence improvement in cardiovascular medication was associated with 93 cardiovascular cases and 140 all‐cause death per 1 million individuals per year in absolute risk differences. Especially, the absolute risk differences per 1 million individuals per year were 118 cardiovascular cases and 175 all‐cause death for statins, 129 all‐cause death for antiplatelet, and 117 all‐cause death for ACEI/ARB for a 20% cardiovascular medication adherence improvement.

### 3.6 Sensitivity analyses and publication bias

The omission of any single study did not significantly alter the pooled RRs. There was no statistical evidence of publication bias (Supporting Information: Figure S1).

## 4 DISCUSSION

To the best of our knowledge, this is the first meta‐analysis that quantitively analyzes the effect of cardiovascular medication adherence on outcomes among patients with CAD in a real‐world setting. By incorporating 18 prospective studies with 402 201 patients, we novelty found a 20% cardiovascular medication adherence unit increased could reduce the risk for cardiovascular events by 8% and all‐cause mortalities by 12%, respectively. Furthermore, by using incidence rates from the general US population, we found the absolute risk difference associated with per 20% medication adherence to cardiovascular medication was 93 per 1 million cardiovascular cases per year and 140 per 1 million all‐cause death. This meta‐analysis strengthens and extends the understanding of the positive impact of cardiovascular medication adherence on secondary prevention among people with CAD, further supporting the notion that improved cardiovascular medication adherence was associated with better outcomes in patients with CAD.

Medication adherence has been defined as the extent to which a patient takes medications as prescribed by their healthcare providers.^30^ In clinical practice, medication nonadherence is one of the main factors that reduce the effectiveness of drug therapies.^31^ However, a previous study reported that almost 40% of the patients who initiated the use of ACEIs/ARBs, beta‐blockers, or statins following hospitalization for MI became nonadherent during the first treatment year.^32^ Moreover, many patients seemed to do so already during the first 6 months.^5^ Therefore, better adherence to cardiovascular treatment should be highlighted in the clinical secondary prevention in patients with CAD.

We subsequently found the inverse association between cardiovascular medication adherence and outcomes in patients with CAD risk was found. A 20% improvement in cardiovascular medication adherence was associated with an 8% reduction in cardiovascular case risk. In addition, this cardiovascular event risk reduction would be translated into a reduction of all‐cause mortality (decreased by 12%). However, this benefit was not observed in good beta‐blocker adherence. This reason might be the majority of the included sample were patients with post‐MI. Recently, several studies reported that beta‐blocker might have no benefit on post‐AMI patients without heart failure or ventricular dysfunction (so‐called reperfusion era).^33^, ^34^ Actually, the evidence of benefits associated with beta‐blocker use is mostly from trials predating the advent of the era of early revascularization.^35^ A meta‐analysis of randomized clinical trials also showed that beta‐blocker use has no mortality benefit for patients without heart failure.^36^ Another study also showed patients adherent to ACE inhibitors/ARBs and statins only had similar mortality rates as those adherents to ACE inhibitors/ARBs and statins and beta‐blocker,^17^ suggesting a limited additional benefit for beta‐blockers in patients with post‐MI. By combining evidence from observational studies, a systematic review suggested that the majority of the included studies failed to demonstrate a benefit in survival or cardiovascular events with long‐term beta‐blockers in post‐MI patients with normal left ventricular function.^37^ However, because of a lack of or limited data, subgroup analysis by subtypes of heart failure was not available. Further studies are needed to explore the impact of beta‐blockers adherence on the outcomes in post‐MI patients (especially in patients with normal left ventricular function).

In general, the impact of age on “statin adherence” in the secondary prevention of CAD remains controversial. Actually, statins are generally not sufficient to recommend among older patients (aged ≥75) with known CVD by current guidelines. The 2013 ACC/AHA guideline^38^ recommend and the US Preventive Services Task Force did not recommend it.^39^ A recent meta‐analysis of RCTs showed significant reductions in major coronary events in all age groups (including those older than 75 years) among participants with known vascular disease.^40^ However, in a propensity‐adjusted analysis of real‐world data, Rothschild et al.^41^ found statin therapy had no significant effect on long‐term survival in older adults hospitalized patients with CAD, but without adjustment for medication adherence. In contrast, another observational study based on patients withatherosclerotic CVD found high‐intensity statins were associated with a small but significant survival advantage compared with moderate‐intensity statins (hazard ratio [HR], 0.90; 95% CI, 0.87–0.94) compared with those receiving submaximal doses.^42^ In the current study, we found that good adherence was associated with reduced mortality in patients with CAD regardless of age (<65 or >65 years), which suggested the benefit of statins persisted in older patients with CAD. However, due to the scarcity of data, we can't perform a subgroup analysis based on sex over 75 years. Therefore, the benefit of the effect of good adherence to statin in CAD patients with older age needs to be further investigated.

The exposure‐effect relationship emphasizes the importance of medication adherence. For example, numerous studies have reported that patients taking a statin before undergoing an MI event is more prone not adherence to a statin, since they may believe that statin therapy is ineffective in preventing future events,^14^ despite experiencing an MI event should not necessarily be viewed as a failure of statin therapy. To date, the effect of good adherence to cardiovascular medications has been validated.^39^, ^40^, ^43^ For example, over a period of 4 years of statin use results in a reduction of 1 mmol/L (39 mg/dl) in the level of low‐density lipoprotein cholesterol, which translates into a 13% reduction in death from all‐cause death.^44^ Some authors have raised the idea of “it is not too late to improve statin adherence” among patients whether they were adherent to statin therapy pre‐AMI.^14^ We also suggest clinicians should emphasize this idea in health propaganda education when patients discharging from the hospital. Besides, poor adherence was affected by lots of factors (i.e., sex, age, race).^45^ Another simple method to solve this problem is to develop predictive screen models to better identify at‐risk for nonadherence patients prospectively. On the other hand, we suggest physicians pay more attention to these patients with a high‐risk factor of nonadherence (e.g., older, depression, cognitive dysfunction).^46^, ^47^ Also, the doctors should improve the communication between doctors and patients or their family and the health‐related beliefs of patients, which has been shown to contribute to patient adherence. Furthermore, some innovations aid can be used to improve adherence management, such as pill‐boxes, calendars, and mobile applications.^48^, ^49^

## 5 LIMITATION

We acknowledged that some limitations exist in this study. First, most research included in this meta‐analysis was focused on statins, the number of studies about beta‐blockers, antiplatelet, and ACEI/ARB is limited, resulting in some subgroup analyses for each drug were not available. Second, because of data restriction on medication doses, we could not estimate what proportion of the risk of cardiovascular events and/or all‐cause death that has been attributed in this review to poor adherence is in fact explained by the prescription of suboptimal medication doses. Third, some studies only defined “regular use” and “no use” adherence without an accurate exposure of adherence and were, therefore, unable to describe the exposure‐effect relationship. So a part of published studies was excluded from current studies, which might cause a selection bias. Lastly, a number of studies showed cardiovascular medication discontinuation is associated with an increased risk of all‐cause death in CAD patients. For example, Ho et al.^50^ showed statin discontinuation is associated with a 2.86‐fold increased risk of mortality (HR, 2.86; 95% CI, 1.47–5.55) over the 1‐year study; however, because of data‐type restriction, the exposure‐effect analysis was not available.

## 6 CONCLUSION

In summary, poor adherence is dose‐dependently associated with significantly increased risk of cardiovascular events and all‐cause mortality in patients with CAD. Cardiovascular medication adherence should be a target for quality improvement interventions to maximize the outcomes of secondary prevention of CAD.

## AUTHOR CONTRIBUTIONS

Kui Hong was responsible for the entire project and revised the draft. Chen Chen and Xiaoqing Li performed the systematic literature review and drafted the first version of the manuscript. All authors take responsibility for all aspects of the reliability and freedom from bias of the data presented and their discussed interpretation.

## CONFLICT OF INTEREST

The authors declare no conflict of interest.

## Supporting information

Supplementary information.

## Data Availability

The data that support the findings of this study are available from the corresponding author upon reasonable request.
