# Supplementary material for: Adherence with cardiovascular medications and the outcomes in patients with coronary arterial disease: “Real‐world” evidence
Source: Clin Cardiol. 2022 Sep 18;45(12):1220–8. doi: 10.1002/clc.23898 (PMC9748759; doi:10.1002/clc.23898)
Supplement: Supplementary file 1 — Supplementary information. [file CLC-45-1220-s001.docx]

**SUPPLEMENTAL MATERIALS**

## ---------------------------------------------------------------------------------------------------------------------------------------------------------------------------------------------

**Adherence with cardiovascular medications and the outcomes in patients with coronary heart disease: ‘real-world’ evidence**

Running title: Medications adherence in CAD

Chen Chen^1, #^, M.D., Xiaoqing Li^1, #^, M.D., Yuhao Su^1^, M.D., Zhigang You^1^, M.D., Ph.D. Rong Wan^2^, Ph.D. Kui Hong^1, 2,^ ^3^***** M.D., Ph.D.

Chen Chen and Xiaoqing Li are co-first authors

&Corresponding Author: Dr. Kui Hong, M.D., Ph.D.

Email: hongkui88@163.com

## Supplemental Methods

## ---------------------------------------------------------------------------------------------------------------------------------------------------------------------------------------------

## Literature Search

This study was conducted according to the recommendations of the PRISMA 2009 guidelines. The Cochrane Library, PubMed, and Embase database up to March 1, 2020, for eligible studies were searched using wide search terms. **Table S2** provides a detailed description of the search strategy. In addition, we searched the reference lists of previous reviews^1, 2^. Our study was registered with PROSPERO (International prospective register of systematic reviews)—registration number—CRD 42019116748.

## Study Selection

Studies were considered eligible if they met the following criteria: 1) included patients with coronary arterial disease; 2) designed as prospective observational studies, including cohort studies or nested case-control studies; 3) the exposure of interest was adherence to statin, anti-platelet agent(aspirin, clopidogrel, etc.), ACEI/ARB, and beta-blockers; 4) assessed outcomes that all-cause death, and cardiovascular death or MI;. 5) reported the relative risk (RR) and the corresponding 95% confidence interval (CI) (or these data could be estimated). RCT trials, cross-sectional studies, reviews, editorials, letters, and animal studies were excluded from our study. For multiple publications/reports based on the same population, the most informative or recent study was used. Studies were excluded if they failed to meet any criteria detailed above. There is no language restriction in this search.

## Data Extraction, quality Assessment, and statistical analyses

Two researchers (C.C. and X.L.) independently worked in the whole process of this meta-analysis from literature search and selection to data analysis. The following data were extracted from each study: first author, publication year, country, age, duration of follow-up, study design, adherence level, assessment method, medication types, RR and 95%CI from the most fully adjusted model for each category. According to previously reported standard definitions, medication adherence was mainly assessed by quantifying the adherence level or determining whether statins were persistently taken during the treatment period. PDC and MPR were similarly obtained by calculating the percentage of days exposed to statins in a given follow-up period^3, 4^. Therefore, PDC and MPR were considered equally for the evaluation of statin adherence^3^. To take a single and fixed exposure assessment, MPR was considered equal to PDC in dose-response analysis.Thus, a study defied adherence level as irregular user, regular user was quantified as poor adherence or good adherence. All discrepancies were resolved through discussion with each other or through consultation with a 3rd reviewer (Y.S.). A nine-star system based on the Newcastle Ottawa scale (NOS) was used to assess study quality. Score over six was defined as a high-quality study. We used the robust error meta-regression method (REMR) described by Xu et al.^5^ for the dose-response analysis. This method is based on a “one-stage approach” which treating each study as a cluster of the whole sample and considering the within-study correlations by clustered robust error. The method requires known levels of medication adherence and RRs with variance estimates for at least two quantitative exposure categories. A method described by *Hamling et a^6^l* was used to transformed the data that did not set the lowest medication adherence group as a reference. If these data could not be obtained from an article, the evidence was not pooled. If the median or mean medication adherence was not provided and reported in ranges, we estimated the midpoint of each category by averaging the lower and upper boundaries of that category. If the highest or lowest category was open-ended, we assumed that the open-ended interval length was the same as the adjacent interval. A P value < 0.05 was considered statistically significant.

## Supplemental Tables

## ---------------------------------------------------------------------------------------------------------------------------------------------------------------------------------------------

**Table S1.** Prisma Checklist 2009

| **Section/topic** | | **#** | | **Checklist item** | | **Reported on page #** | |
| --- | --- | --- | --- | --- | --- | --- | --- |
| **TITLE** | | | | | |  | |
| Title | | 1 | | Identify the report as a systematic review, meta-analysis, or both. | | 1-2 | |
| **ABSTRACT** | | | | | |  | |
| Structured summary | | 2 | | Provide a structured summary including, as applicable: background; objectives; data sources; study eligibility criteria, participants, and interventions; study appraisal and synthesis methods; results; limitations; conclusions and implications of key findings; systematic review registration number. | | 3-4 | |
| **INTRODUCTION** | | | | | |  | |
| Rationale | | 3 | | Describe the rationale for the review in the context of what is already known. | | 5 | |
| Objectives | | 4 | | Provide an explicit statement of questions being addressed with reference to participants, interventions, comparisons, outcomes, and study design (PICOS). | | 5 | |
| **METHODS** | | | | | |  | |
| Protocol and registration | | 5 | | Indicate if a review protocol exists, if and where it can be accessed (e.g., Web address), and, if available, provide registration information including registration number. | | 6 | |
| Eligibility criteria | | 6 | | Specify study characteristics (e.g., PICOS, length of follow-up) and report characteristics (e.g., years considered, language, publication status) used as criteria for eligibility, giving rationale. | | 6 | |
| Information sources | | 7 | | Describe all information sources (e.g., databases with dates of coverage, contact with study authors to identify additional studies) in the search and date last searched. | | 6 | |
| Search | | 8 | | Present full electronic search strategy for at least one database, including any limits used, such that it could be repeated. | | 6 | |
| Study selection | | 9 | | State the process for selecting studies (i.e., screening, eligibility, included in systematic review, and, if applicable, included in the meta-analysis). | | 7 | |
| Data collection process | | 10 | | Describe method of data extraction from reports (e.g., piloted forms, independently, in duplicate) and any processes for obtaining and confirming data from investigators. | | 7 | |
| Data items | | 11 | | List and define all variables for which data were sought (e.g., PICOS, funding sources) and any assumptions and simplifications made. | | 7 | |
| Risk of bias in individual studies | | 12 | | Describe methods used for assessing risk of bias of individual studies (including specification of whether this was done at the study or outcome level), and how this information is to be used in any data synthesis. | | 7 | |
| Summary measures | | 13 | | State the principal summary measures (e.g., risk ratio, difference in means). | | 8 | |
| Synthesis of results | | 14 | | Describe the methods of handling data and combining results of studies, if done, including measures of consistency (e.g., I^2^) for each meta-analysis. | | 8 | |
| Risk of bias across studies | | 15 | | Specify any assessment of risk of bias that may affect the cumulative evidence (e.g., publication bias, selective reporting within studies). | | 8 | |
| Additional analyses | | 16 | | Describe methods of additional analyses (e.g., sensitivity or subgroup analyses, meta-regression), if done, indicating which were pre-specified. | | 8 | |
| **RESULTS** | | | | |  | |  |
| Study selection | 17 | | Give numbers of studies screened, assessed for eligibility, and included in the review, with reasons for exclusions at each stage, ideally with a flow diagram. | | 9 | |  |
| Study characteristics | 18 | | For each study, present characteristics for which data were extracted (e.g., study size, PICOS, follow-up period) and provide the citations. | | 9 | |  |
| Risk of bias within studies | 19 | | Present data on risk of bias of each study and, if available, any outcome level assessment (see item 12). | | 11 | |  |
| Results of individual studies | 20 | | For all outcomes considered (benefits or harms), present, for each study: (a) simple summary data for each intervention group (b) effect estimates and confidence intervals, ideally with a forest plot. | | 8 | |  |
| Synthesis of results | 21 | | Present results of each meta-analysis done, including confidence intervals and measures of consistency. | | 8 | |  |
| Risk of bias across studies | 22 | | Present results of any assessment of risk of bias across studies (see Item 15). | | 10 | |  |
| Additional analysis | 23 | | Give results of additional analyses, if done (e.g., sensitivity or subgroup analyses, meta-regression [see Item 16]). | | 10-11 | |  |
| **DISCUSSION** | | | | |  | |  |
| Summary of evidence | 24 | | Summarize the main findings including the strength of evidence for each main outcome; consider their relevance to key groups (e.g., healthcare providers, users, and policy makers). | | 11 | |  |
| Limitations | 25 | | Discuss limitations at study and outcome level (e.g., risk of bias), and at review-level (e.g., incomplete retrieval of identified research, reporting bias). | | 14 | |  |
| Conclusions | 26 | | Provide a general interpretation of the results in the context of other evidence, and implications for future research. | | 15 | |  |
| **FUNDING** | | | | |  | |  |
| Funding | 27 | | Describe sources of funding for the systematic review and other support (e.g., supply of data); role of funders for the systematic review. | | 15 | |  |

**Table S2:** Search strategy
PubMed database

| Search Terms | Search Options | Results |
| --- | --- | --- |
| #1 | adherence | 237,011 |
| #2 | compliance | [176,860](https://www.ncbi.nlm.nih.gov/pubmed/?cmd=HistorySearch&querykey=3) |
| #3 | asprin | 64,677 |
| #4 | clopidogrel | [13,787](https://www.ncbi.nlm.nih.gov/pubmed/?cmd=HistorySearch&querykey=5) |
| #6 | angiotensin-converting enzyme inhibitors | 50,800 |
| #7 | angiotensin receptor blockers | 33,125 |
| #8 | beta-blockers | [98,167](https://www.ncbi.nlm.nih.gov/pubmed/?cmd=HistorySearch&querykey=6) |
| #9 | statin | [54,336](https://www.ncbi.nlm.nih.gov/pubmed/?cmd=HistorySearch&querykey=7) |
| #10 | hydroxymethylglutaryl-CoA reductase inhibitors | 39,639 |
| #11 | Cardiovascular disease | 2,127,330 |
| #12 | death | [908,674](https://www.ncbi.nlm.nih.gov/pubmed/?cmd=HistorySearch&querykey=9) |
| #13 | mortality | [119,5170](https://www.ncbi.nlm.nih.gov/pubmed/?cmd=HistorySearch&querykey=10) |
| #17 | #1 OR #2 | 43,057 |
| #18 | #3 OR #4 OR #5 OR #6 OR #7 OR #8 OR #9 OR #10 | 220,682 |
| #19 | #11 OR #12 OR #13 OR #14 OR #15 | 3949,050 |
| #20 | #17 AND #18 AND #19 | 454 |

**Cochrane library**

| Search Terms | Search Options | Results |
| --- | --- | --- |
| #1 | adherence | 2,199 |
| #2 | compliance | 2,630 |
| #3 | asprin | 496 |
| #4 | clopidogrel | 77 |
| #5 | antiplatelet | 268 |
| #6 | angiotensin-converting enzyme inhibitors | 193 |
| #7 | angiotensin receptor blockers | 140 |
| #8 | beta-blockers | 235 |
| #9 | statin | 195 |
| #10 | hydroxymethylglutaryl-CoA reductase inhibitors | 36 |
| #11 | Cardiovascular disease | 1,713 |
| #12 | death | 4,766 |
| #13 | mortality | 4,187 |
| #14 | #1 OR #2 | 1,046 |
| #15 | #3 OR #4 OR #5 OR #6 OR #7 OR #8 OR #9 OR #10 | 519 |
| #16 | #11 OR #12 OR #13 | 5,845 |
| #17 | #14 AND #15 AND #16 | 80 |

**Embase database**

| Search Terms | Search Options | Results |
| --- | --- | --- |
| #1 | adherence | 196,514 |
| #2 | compliance | 313,456 |
| #3 | aspirin | 115,727 |
| #4 | clopidogrel | 59,326 |
| #5 | antiplatelet | 45,128 |
| #6 | angiotensin-converting enzyme inhibitors | 111,419 |
| #7 | angiotensin receptor blockers | 39,391 |
| #8 | beta-blockers | 22,768 |
| #9 | statin | 39,510 |
| #10 | hydroxymethylglutaryl-CoA reductase inhibitors | 83,763 |
| #11 | Cardiovascular disease | 369,651 |
| #12 | death | 1,214,059 |
| #13 | mortality | 1,426,759 |
| #17 | #1 OR #2 | 61,720 |
| #18 | #3 OR #4 OR #5 OR #6 OR #7 OR #8 OR #9 OR #10 | 174,173 |
| #19 | #11 OR #12 OR #13 | 2,899,797 |
| #20 | #17 AND #18 AND #19 | 718 |

**Table S3**. Quality assessment of included studies

| Author  (Publication Year) | Newcastle-Ottawa Scale | | | | | | | | | |
| --- | --- | --- | --- | --- | --- | --- | --- | --- | --- | --- |
|  | Selection | | | Comparability | | | Outcome | | | Total |
|  | a | b | c | d | e | f | g | h | i |  |
| Allonen 2012 | 1 | 1 | 1 | 1 | 1 | 1 | 1 | 0 | 0 | 7 |
| Hamood 2015 | 1 | 1 | 1 | 1 | 1 | 1 | 1 | 1 | 0 | 8 |
| Ho 2006 | 1 | 1 | 1 | 1 | 0 | 1 | 1 | 1 | 1 | 8 |
| Kleiner, 2009 | 1 | 1 | 1 | 1 | 1 | 1 | 1 | 0 | 0 | 7 |
| Lenzi 2014 | 1 | 1 | 1 | 1 | 0 | 1 | 1 | 1 | 0 | 7 |
| Martino 2015 | 1 | 1 | 1 | 1 | 0 | 1 | 1 | 1 | 0 | 7 |
| Rasmussen 2007 | 1 | 1 | 1 | 1 | 1 | 1 | 1 | 1 | 1 | 9 |
| Rublee 2012 | 1 | 1 | 1 | 1 | 0 | 1 | 1 | 0 | 0 | 6 |
| Ruokoniemi,2011 | 1 | 1 | 1 | 1 | 1 | 1 | 1 | 1 | 0 | 8 |
| Tuppin 2010 | 1 | 1 | 1 | 1 | 1 | 1 | 1 | 1 | 0 | 8 |
| Shalev 2009 | 1 | 1 | 1 | 1 | 1 | 1 | 1 | 0 | 0 | 7 |
| Wei 2002 | 1 | 1 | 1 | 1 | 1 | 1 | 1 | 1 | 0 | 8 |
| WEI 2008 | 1 | 1 | 1 | 1 | 1 | 1 | 1 | 1 | 0 | 8 |
| Wei 2004 | 1 | 1 | 1 | 1 | 1 | 1 | 1 | 1 | 0 | 8 |
| Xie, 2017 | 1 | 1 | 1 | 1 | 1 | 1 | 1 | 1 | 1 | 9 |
| McGinnis, 2009 | 1 | 1 | 1 | 1 | 1 | 1 | 1 | 1 | 0 | 8 |
| Hickson, 2019 | 1 | 1 | 1 | 1 | 1 | 1 | `1 | 1 | 1 | 9 |
| korhonen,2017 | 1 | 1 | 1 | 1 | 1 | 1 | 1 | 1 | 0 | 8 |

1. Representativeness of the exposed cohort.
2. Selection of the non-exposed cohort.
3. Ascertainment of exposure.
4. Demonstration that outcome of interest was not present at start of study.
5. Comparability of cohorts on the basis of the design or analysis (adjusted for age).
6. Comparability of cohorts on the basis of the design or analysis (adjusted for any other factor).
7. Assessment of outcome.
8. Was follow-up long enough for outcomes to occur..
9. Adequacy of follow-up of cohorts.

**Table S4.** Subgroup analysis-cardiovascular medication adherence and all-cause death

| Items | | Number of studies | RR (95%CI)  Per 20% adherence increase | *I*^2^(%) | Within subgroup | P _non-linear_ |
| --- | --- | --- | --- | --- | --- | --- |
| Age | <65 years | 3 | 0.83(0.829-0.84) | 99 | <0.001 | 0.59 |
|  | >65 years | 12 | 0.94(0.90-0.98) | 0 | 0.01 | 0.11 |
| Region | Northern America | 6 | 0.93(0.90-0.96) | 62 | 0.002 | 0.69 |
|  | Europe | 6 | 0.84(0.83-0.85) | 99 | <0.001 | 0.69 |
|  | Asia | 3 | 0.84(0.78-0.91) | 98 | 0.01 | 0.35 |
| Mean follow-up duration | < 2 years | 6 | 0.87(0.79-0.97) | 56 | 0.02 | 0.50 |
|  | > 2 years | 9 | 0.90(0.84-0.97) | 0 | 0.008 | 0.07 |
| Population | Post-MI | 9 | 0.95(0.91-0.99) | 74 | 0.02 | 0.21 |
|  | Others | 6 | 0.84(0.83-0.85) | 99 | <0.001 | 0.62 |
| Sample size | < 10 000 | 10 | 0.86(0.81-0.90) | 79 | <0.001 | 0.29 |
|  | > 10 000 | 5 | 0.89(0.80-0.98) | 43 | 0.03 | 0.02 |
| Medication types | Statin | 13 | 0.85(0.82-0.89) | 82 | <0.001 | 0.23 |
|  | Anti-platelet | 3 | 0.89(0.85-0.92) | 97 | 0.002 | NA |
|  | ACEI/ARB | 5 | 0.90(0.80-1.00) | 47 | 0.05 | 0.25 |
|  | β-blocker | 8 | 0.97(0.93-1.03) | 97 | 0.32 | 0.22 |

## Supplemental Figures

**Figure S1:** Funnel plots showing associations of major medication groups with cornory artery diseases and cardiovascular events or all-cause death


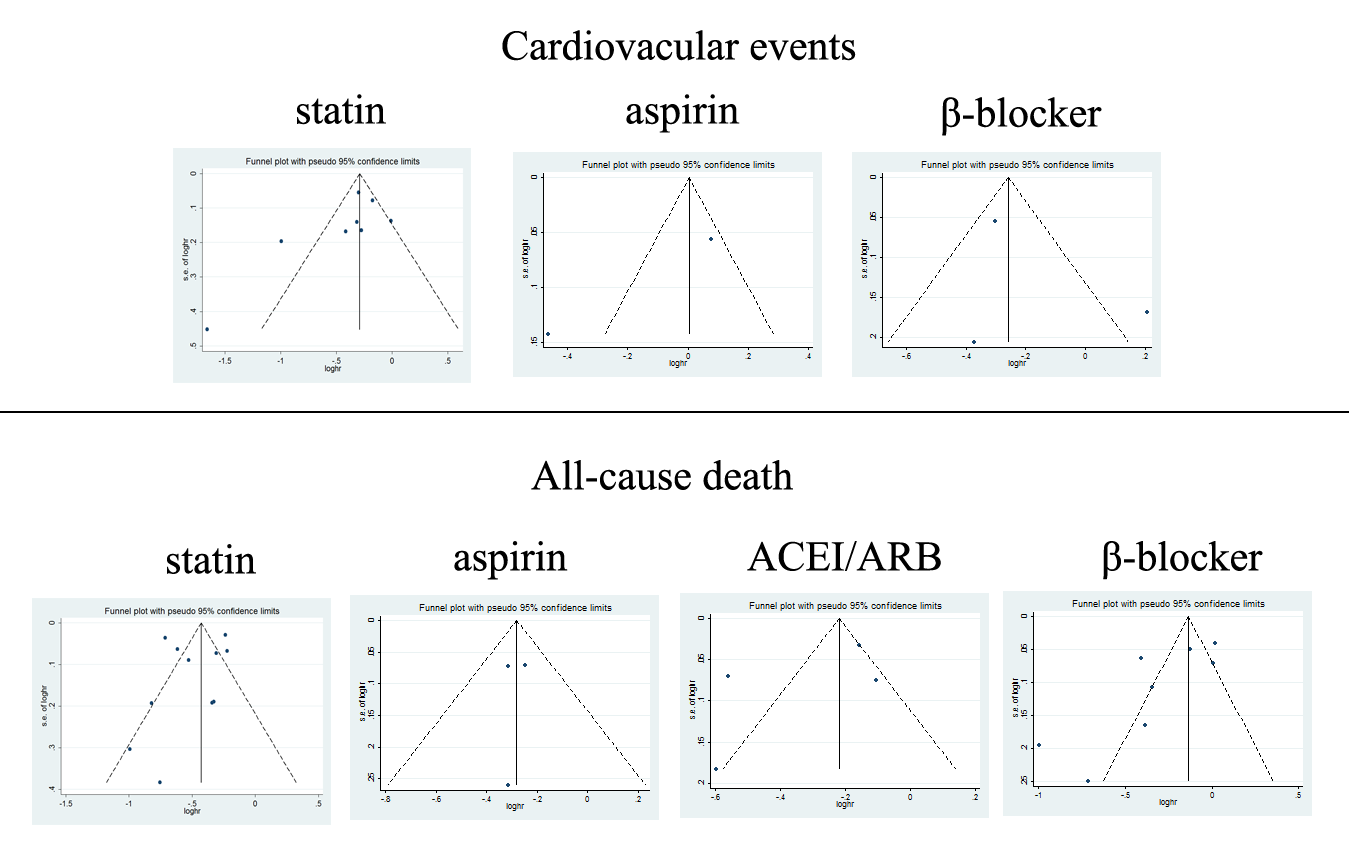


**Reference**

**1.** Dahl Aarvik M, Sandven I, Dondo TB, et al. Effect of oral β-blocker treatment on mortality in contemporary post-myocardial infarction patients: a systematic review and meta-analysis. *European Heart Journal–Cardiovascular Pharmacotherapy.* 2018;5:12-20.

**2.** Ho PM, Bryson CL, Rumsfeld JS. Medication adherence: its importance in cardiovascular outcomes. *Circulation.* 2009;119:3028-3035.

**3.** Lavikainen P, Helin-Salmivaara A, Eerola M, et al. Statin adherence and risk of acute cardiovascular events among women: a cohort study accounting for time-dependent confounding affected by previous adherence. *BMJ open.* 2016;6:e011306.

**4.** Bijlsma MJ, Janssen F, Hak E. Estimating time‐varying drug adherence using electronic records: extending the proportion of days covered (PDC) method. *Pharmacoepidemiology and drug safety.* 2016;25:325-332.

**5.** Xu C, Liu Y, Jia PL, et al. The methodological quality of dose-response meta-analyses needed substantial improvement: a cross-sectional survey and proposed recommendations. *J Clin Epidemiol.* 2019;107:1-11.

**6.** Hamling J, Lee P, Weitkunat R, Ambuhl M. Facilitating meta-analyses by deriving relative effect and precision estimates for alternative comparisons from a set of estimates presented by exposure level or disease category. *Stat Med.* 2008;27:954-970.
